# Impact of Exercise on Cardiometabolic Component Risks in Spinal Cord–injured Humans

**DOI:** 10.1249/MSS.0000000000001390

**Published:** 2017-11-14

**Authors:** TOM E. NIGHTINGALE, JEAN-PHILIPPE WALHIN, DYLAN THOMPSON, JAMES L. J. BILZON

**Affiliations:** Department for Health, University of Bath, Bath, Somerset, UNITED KINGDOM

**Keywords:** GENE EXPRESSION, INSULIN SENSITIVITY, PARAPLEGIA, MODERATE-INTENSITY EXERCISE, METABOLIC HEALTH, CARDIOVASCULAR FITNESS

## Abstract

**Purpose:**

Spinal cord injury (SCI) creates a complex pathology, characterized by low levels of habitual physical activity and an increased risk of cardiometabolic disease. This study aimed to assess the effect of a moderate-intensity upper-body exercise training intervention on biomarkers of cardiometabolic component risks, adipose tissue metabolism, and cardiorespiratory fitness in persons with SCI.

**Methods:**

Twenty-one inactive men and women with chronic (>1 yr) SCI (all paraplegic injuries) 47 ± 8 yr of age (mean ± SD) were randomly allocated to either a 6-wk prescribed home-based exercise intervention (INT; *n* = 13) or control group (CON; *n* = 8). Participants assigned to the exercise group completed 4 × 45-min moderate-intensity (60%–65% peak oxygen uptake (V˙O_2peak_)) arm-crank exercise sessions per week. At baseline and follow-up, fasted and postload blood samples (collected during oral glucose tolerance tests) were obtained to measure metabolic regulation and biomarkers of cardiovascular disease. Abdominal subcutaneous adipose tissue biopsies were also obtained, and cardiorespiratory fitness was assessed.

**Results:**

Compared with CON, INT significantly decreased (*P* = 0.04) serum fasting insulin (Δ, 3.1 ± 10.7 pmol·L^−1^ for CON and −12.7 ± 18.7 pmol·L^−1^ for INT) and homeostasis model assessment of insulin resistance (HOMA2-IR; Δ, 0.06 ± 0.20 for CON and −0.23 ± 0.36 for INT). The exercise group also increased V˙O_2peak_ (Δ, 3.4 mL·kg^−1^·min^−1^; *P* ≤ 0.001). Adipose tissue metabolism, composite insulin sensitivity index (C-ISI_Matsuda_), and other cardiovascular disease risk biomarkers were not different between groups.

**Conclusions:**

Moderate-intensity upper-body exercise improved aspects of metabolic regulation and cardiorespiratory fitness. Changes in fasting insulin and HOMA2-IR, but not C-ISI_Matsuda_, suggest improved hepatic but not peripheral insulin sensitivity after 6 wk of exercise training in persons with chronic paraplegia.

Epidemiological studies suggest that mortality and morbidity rates associated with cardiovascular disease and Type 2 diabetes are elevated in individuals with spinal cord injury (SCI) relative to adults without physical disabilities (11,19). Risk factors for developing these chronic diseases, including increased central adiposity (9), reduced HDL cholesterol (13), and impaired glucose tolerance (2), occur at a heightened frequency in this population. One modifiable lifestyle factor, widely accepted to influence the incidence of these noncommunicable diseases in the general population and often not performed regularly in persons with SCI (3), is physical activity. It is therefore intuitive to suggest that exercise may play a beneficial role in improving cardiometabolic component risks in the SCI population.

Although upper-body aerobic exercise in this population improves strength and cardiorespiratory fitness, its effect on cardiometabolic component risks in adults with SCI remains unclear (4). Adhering to the current Physical Activity Guidelines for Spinal Cord Injury (PAG-SCI: >40 min min·wk^−1^ moderate- to vigorous-intensity aerobic exercise) failed to improve biomarkers of cardiovascular disease (8). With voluntary movements restricted to the smaller upper-body skeletal muscles of persons with SCI, achieving the same energy expenditure as moderate-intensity whole-body exercise might require a greater volume of exercise to stimulate similar physiological adaptations. Whether a larger volume of exercise, above that recommended for the general population, is capable of improving cardiometabolic component risks in persons with SCI has yet to be established.

Evidence suggests that two of three people with SCI are obese (12). Given its role in lipid storage and its function as an endocrine organ (36), adipose tissue may play a prominent role in regulating whole-body adaptations to exercise training in persons with SCI. Animal models suggest that exercise training results in wide-ranging and multifunctional adaptations to subcutaneous adipose tissue, including increased expression of several key metabolic proteins (33,35). Interestingly, daily vigorous-intensity treadmill running has been shown to counteract the negative effects of short-term weight gain at the whole-body level and in adipose tissue in healthy young men (39). Therefore, exercise may also have beneficial effects on whole-body and adipose tissue metabolism in persons with SCI. Exercise interventions may serve as a preventive measure in SCI patients, reducing the burden of long-term chronic disease in this population.

The aim of this study was to determine the effect of moderate-intensity exercise on cardiometabolic component risks, the expression of key genes within adipose tissue, and cardiorespiratory fitness in persons with chronic paraplegia. We hypothesized that, in comparison to a control group, 6 wk of home-based moderate-intensity exercise would improve (i) metabolic regulation, (ii) biomarkers of cardiovascular disease, (iii) adipose tissue metabolism, and (iv) cardiorespiratory fitness in persons with SCI.

## METHODS

**FIGURE 1:**
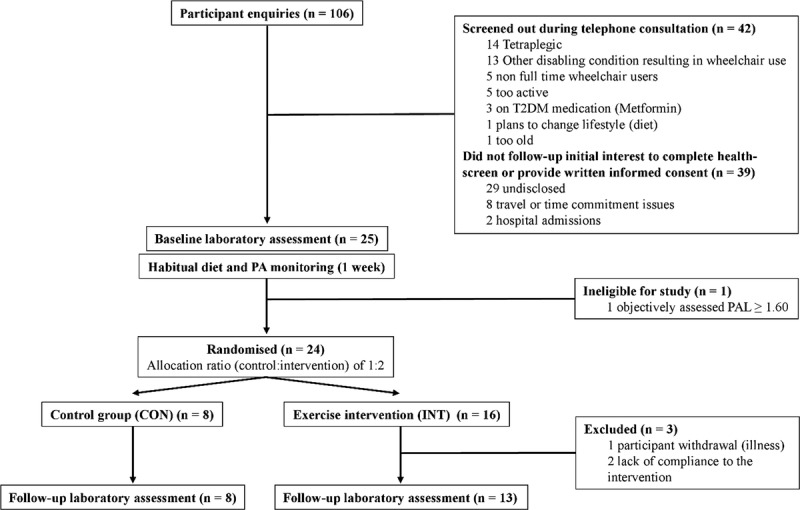
CONSORT flow diagram for HOMEX-SCI trial.

HOMEX-SCI (ISRCTN57096451) is a randomized controlled trial that compares the effect of moderate-intensity upper-body home-based exercise (INT) relative to a control group (CO) on health indices and adipose tissue metabolism in persons with chronic SCI. The trial protocol was approved by the South West (Exeter) National Research Ethics Service Committee (14/SW/0106), and all participants provided written informed consent. A detailed trial protocol has since been published (26) and is in accordance with current Consolidated Standards of Reporting Trials (CONSORT) guidelines (31) (Fig. 1). The same experimental procedures were performed during both baseline and follow-up laboratory testing.

### 

#### Participants

Study participants who met the following criteria were included in the study: 18–65 yr of age, male or female, inactive (habitual physical activity level (PAL) ≤1.60), with chronic (>1 yr) paraplegia at or below T2, no immediate plans to alter diet and/or physical activity behavior, weight stable (±3 kg during the previous 6 months), free from active medical issues (i.e., pressure sores, urinary tract infections, and cardiovascular contraindications for testing) or musculoskeletal complaints, and not taking antihyperglycemic medication. Participants with neurologically incomplete injuries were considered eligible provided that they used a wheelchair >75% of their waking day. Baseline demographic and anthropometric characteristics of participants who completed the trial are presented in Table 1.

**TABLE 1:**
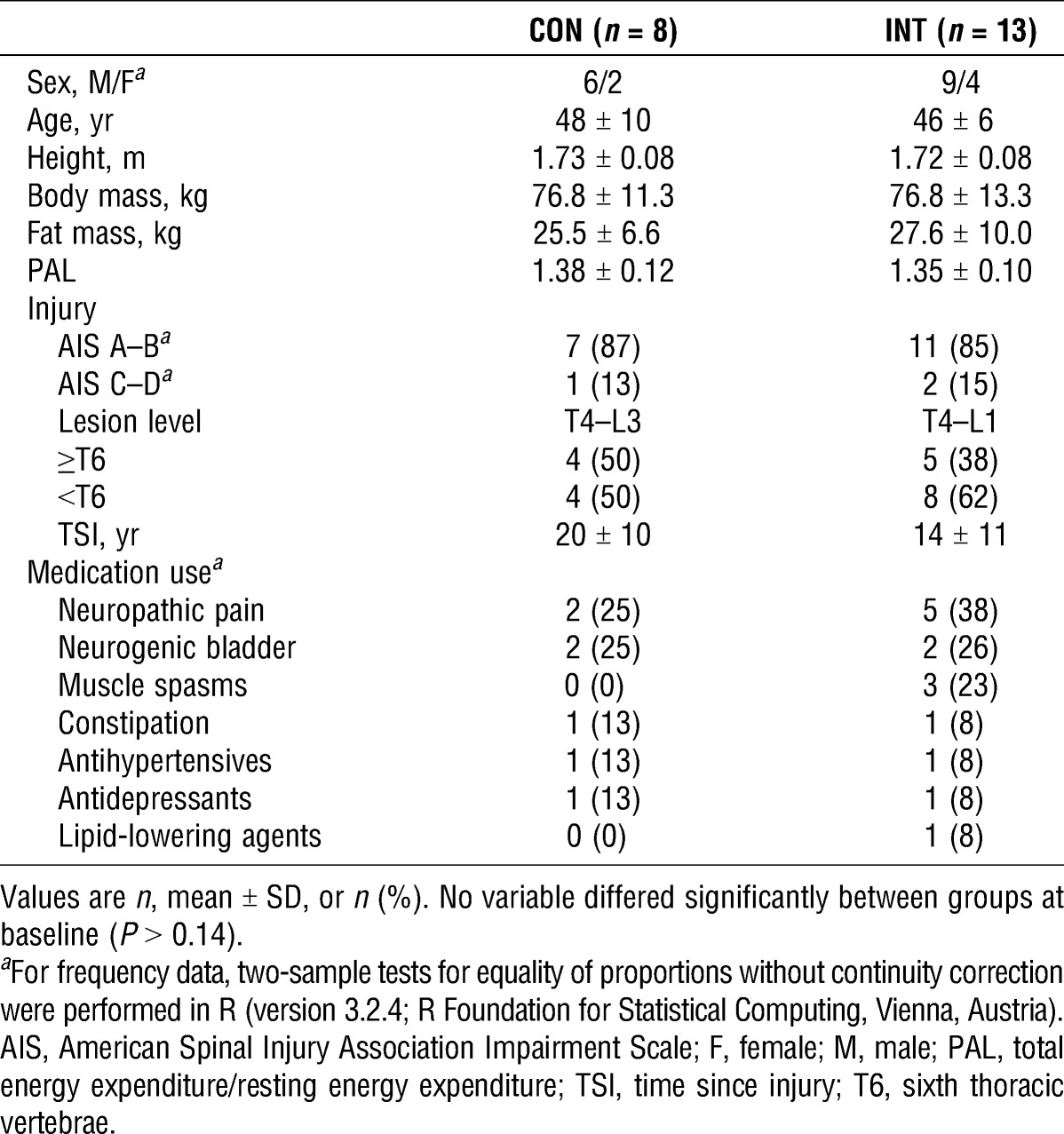
Baseline demographic and anthropometric characteristics.

#### Experimental design and protocol

Anthropometric characteristics are as follows: supine height (Lufkin, Sparks, MD), body mass (Detecto® BRW1000, Webb City, MO), fat mass, lean mass, and visceral adipose tissue area (dual-energy x-ray absorptiometry; Discovery, Hologic, Bedford, UK) were determined at 0830 ± 1 h. Although the participants remained in a 10-h overnight fast, resting metabolic rate was measured in a supine position via indirect calorimetry from expired gasses. A 20-mL blood sample was drawn from an antecubital vein via an indwelling cannula (BD Venflon Pro; BD, Helsingborg, Sweden), with separated samples of serum and plasma immediately cooled on dry ice and stored at −80°C. A small (1–2 g) subcutaneous adipose tissue biopsy performed under local anesthesia (1% lignocaine hydrochloride) was then sampled from the abdomen using an established “needle aspiration” technique. The sample was cleaned with isotonic saline, and any clots were manually removed. Once the sample had been weighed, it was homogenized in 5 mL of TRIzol (Invitrogen, Paisley, UK) and placed on dry ice until being stored at −80°C. After these procedures, an oral glucose tolerance test (OGTT) was performed, whereby the participants ingested 75 g of anhydrous glucose in a Polycal solution (Nutrica, Trowbridge, UK) within 5 min. Once ingested, 5-mL blood samples were collected every 15 min for 2 h. Blood pressure was also measured in triplicate using an automated monitor (Boso Medicus Prestige; Bosch + Sohn GmbH, Jungingen, Germany).

After these resting measurements, participants’ subpeak and peak physiological responses to arm-crank ergometry were assessed. Participants undertook a discontinuous, incremental subpeak test on the same portable desktop ergometer (Monark 871E, Dalarna, Sweden) provided to them during the INT. Peak oxygen uptake (V˙O_2peak_) and workload were measured at the point of volitional exhaustion during a continuous, incremental exercise protocol (26) performed on an electrically braked arm-crank ergometer (Lode Angio, Groningen, the Netherlands). During both of these exercise protocols, expired gasses were continuously analyzed using a calibrated computerized metabolic system (TrueOne 2400®; ParvoMedics, Salt Lake City, UT). Heart rate was also recorded using a heart rate monitor (T31; Polar Electro Inc., Lake Success, NY). Investigators conducting the V˙O_2peak_ test were not blinded to group allocation because of logistical considerations of managing the trial, but verbal encouragement was standardized between baseline and follow-up.

For 7 d after baseline laboratory testing, participants’ free-living habitual energy intake (weighed-food diaries) and energy expenditure (individually calibrated ActiHeart™) were estimated. To estimate energy intake, participants were provided with a set of weighing scales (PL11B; Smart Weigh, Chestnut Ridge, NY) and trained by the experimenters during the baseline trial how to record food intake. Diet records were analyzed using Nutritics software (Nutritics Ltd., Dublin, Ireland), to estimate energy intake and macronutrient composition. To estimate energy expenditure, participants wore a chest-mounted Actiheart™ device (Cambridge Neurotechnology Ltd., Papworth, UK), individually calibrated for each participant using heart rate data collected at rest and during laboratory exercise testing (26). One participant was excluded (PAL >1.60) from the study. During the 6-wk intervention, eligible participants were randomly assigned (2/1 allocation ratio) to home-based moderate-intensity upper-body exercise (INT) or a lifestyle maintenance (CON). Minimization was used to ensure balance between the two groups for baseline characteristics of age, body mass, level of spinal cord lesion, and PAL. The 6-wk intervention period began ~2 wk after baseline laboratory testing to account for free-living assessment; returning, downloading, and analyzing data for participant eligibility; and delivery of portable arm-crank ergometer. During this 2-wk interim period, participants were instructed not to alter their normal activity patterns or dietary behaviors. Consequently, there were ~8 wk between baseline and follow-up testing, ensuring that eumenorrheic (*n* = 3) female participants were in the follicular phase of their menstrual cycle for both laboratory visits. Besides providing the necessary tools for free-living assessment and arranging follow-up testing, investigator contact with the CON group was negligible to minimize any potential effect on participant behavior.

#### Home-based exercise intervention

The INT group performed moderate-intensity exercise four times per week on a portable desktop arm-crank ergometer in their own home. The duration of each exercise session was extended by 5 min per session throughout the first week (i.e., from 30 to 45 min). The exercise intensity was also increased from ~60% V˙O_2peak_ during the first 3 wk to ~65% V˙O_2peak_ for the final 3 wk. The first exercise session was supervised by an experimenter to ensure that the arm-crank ergometer was set up appropriately and the correct duration and intensity of exercise were adhered to. Compliance with subsequent sessions was monitored via a GENEActiv triaxial accelerometer (Activinsights, Cambridge, UK) worn on the wrist. During the final week of the intervention, energy intake and expenditure were estimated again. No dietary constraints were imposed. The last bout of exercise in the INT group was >36 h before follow-up laboratory testing.

#### Blood and adipose tissue analysis

Key systemic metabolites and hormones were measured via commercially available spectrophotometric assays (serum triacylglycerol, total cholesterol and HDL cholesterol, nonesterified fatty acids (NEFA), and plasma glucose from Randox Laboratories, Co., Antrim, UK) and enzyme-linked immunosorbent assays (serum insulin; Mercodia AB, Uppsala, Sweden). Adipose tissue samples were defrosted and the aqueous phase was mixed with an equal volume of 70% ethanol before being loaded onto an RNeasy mini-column for extraction (Qiagen, Crawley, UK). The amount of RNA was quantified using spectrophotometry, with 2 μg of total RNA reverse transcribed using a high-capacity cDNA reverse transcription kit (Applied Biosystems, Warrington, UK). Assays from Applied Biosystems were used, as follows: adipose triglyceride lipase (ATGL; Hs00386101), fatty acid synthase (Hs00188012_m1), hormone-sensitive lipase (Hs00193510), lipoprotein lipase (Hs01012567_m1), sterol regulatory element binding protein 1c (Hs01088691_m1), 5′-AMP–activated protein kinase (AMPK; Hs01562315_m1 and Hs00178903_m1, combined), glucose transporter type 4 (GLUT4; Hs00168966_m1), insulin receptor substrate 2 (Hs00275843_s1), pyruvate dehydrogenase kinase isozyme (Hs00176875_m1), and peroxisome proliferator–activated receptor gamma coactivator 1-alpha (PGC-1α; Hs01016719_m1). Peptidylpropyl isomerase A (PPIA) was used as an endogenous control, with real-time polymerase chain reaction performed using a StepOne™ (Applied Biosystems). Data were processed using the comparative threshold cycle (Ct) method (ΔCt = Ct target gene − Ct endogenous control) and normalized to an internal calibrator and baseline.

#### Statistical analyses

Serial measurements of glucose and insulin responses to the OGTT at baseline and follow-up were converted into simple summary statistics (i.e., within-subject fasting concentrations, incremental area under the curve, and indices of insulin sensitivity/resistance). Responses within and between trials were analyzed by two-way (group–day) mixed-model ANOVA. ANOVA was performed irrespective of any minor deviations from a normal distribution but with Greenhouse–Geisser corrections applied to intraindividual contrasts, where *ɛ* < 0.75, and the Huynh–Feldt corrections applied for less severe asphericity. Where significant interaction effects were observed, paired *t*-tests were applied to determine significant differences within groups. As a subgroup analysis, participants in the INT group were stratified into two groups, injury levels of ≥T6 and <T6, to assess whether cardiometabolic component risk outcomes responded differently to exercise training. Standardized effect sizes (Cohen *d*) were also calculated and reported for variables with significant effects. On the basis of the magnitude of correlation between trials, thresholds of >0.2 (small), >0.5 (moderate), and >0.8 (large) were used. Statistical analysis for the gene expression data was carried out on the log-transformed data after normalization to an internal calibrator and baseline (21). Independent *t*-tests were calculated between groups for all baseline variables. Statistical analyses were performed using SPSS version 22 (IBM, Armonk, NY), with statistical significance accepted at *a priori* of *α* ≤ 0.05. Values are means with SD in text and tables, with effects expressed as change (Δ) scores with 95% confidence intervals (CI).

## RESULTS

Of the 24 participants who were randomized to either the INT (*n* = 16) or CON (*n* = 8) group, 21 participants (13 INT, 8 CON) were retained for data analyses. The reasons for exclusion were illness/infection (*n* = 1) and lack of adherence to the intervention (*n* = 2). In the INT group, 12 of 13 participants completed all 24 training sessions, that is, 100% compliance. One participant missed two nonconsecutive sessions over the course of 6 wk (92% compliance). During the 6-wk period, mean subjective ratings of difficulty for the INT group across all exercise sessions was 7 ± 1 (1, easy; 10, hard), mean exercise session duration was 44 ± 1 min, and mean power output was 46 ± 18 W. Measured mean exercise heart rate was 144 ± 11 bpm.

### 

#### Free-living energy expenditure and intake

The moderate-intensity upper-body exercise intervention significantly (*P* ≤ 0.01) increased physical activity energy expenditure (PAEE) and minutes spent performing moderate-to-vigorous intensity physical activity (≥3 METs) relative to the CON group (Table 2). In the INT group, objectively measured PAEE was 109 ± 85 kcal·d^−1^ higher (*P* ≤ 0.001) relative to baseline. Energy intake and macronutrient composition did not change over time (*P* > 0.2), with no evidence of an interaction effect (*P* > 0.2).

**TABLE 2:**
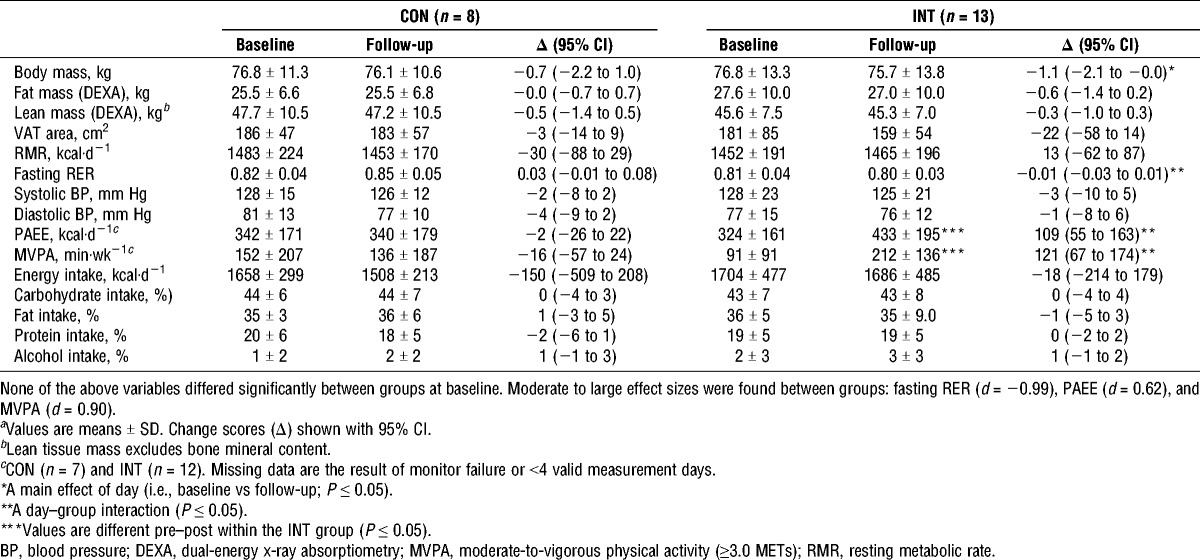
Body composition, resting physiological characteristics, free-living physical activity, and diet components measured at baseline and after 6 wk (follow-up) of CON or INT*^a^*.

#### Body composition

Body composition and resting physiological measures at baseline and follow-up are summarized in Table 2. Body mass significantly decreased from baseline to follow-up when all participants in both groups were considered (*P* ≤ 0.05). The absolute change was not different between the INT (−1.1 kg; 95% CI, −2.1 to −0.0 kg) and CON (−0.7 kg; 95% CI, −2.2 to 1.0 kg) groups (*P* = 0.6). Body mass changes were predominantly explained by reductions in fat mass (−0.6; 95% CI, −1.4 to 0.2 kg) and lean mass (−0.5 kg; 95% CI, −1.4 to 0.5 kg) in the INT and CON groups, respectively.

#### Functional capacity (V˙O_2peak_ and power output)

**FIGURE 2:**
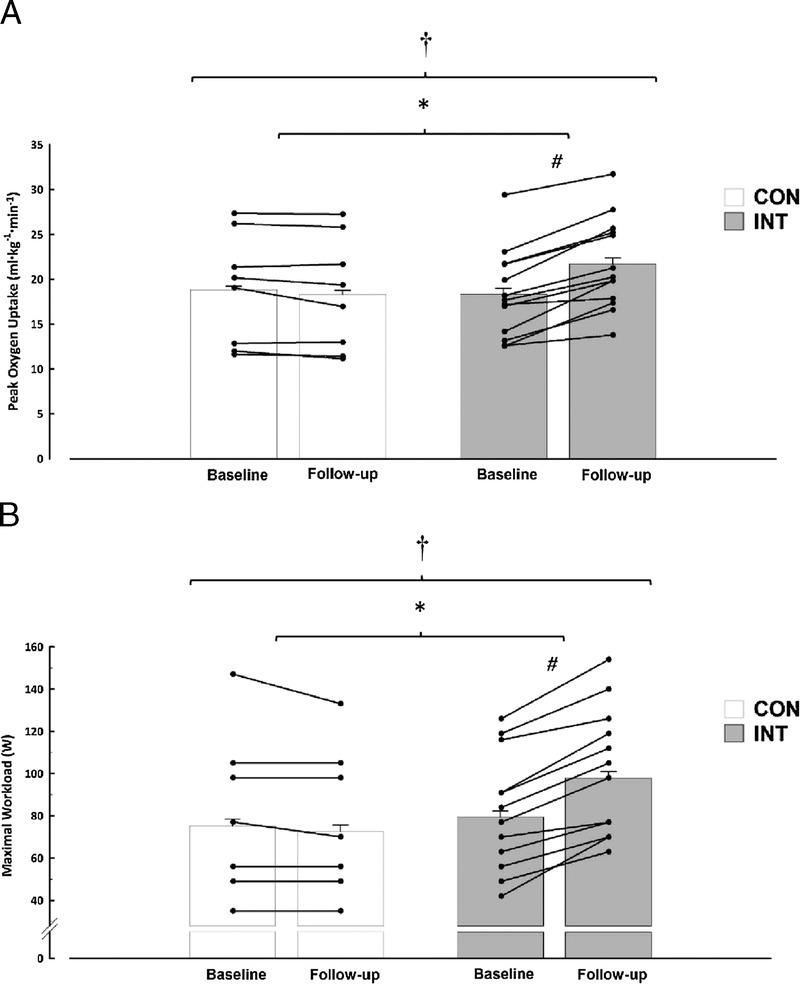
V˙O_2peak_ (A) and workload (B) at baseline and after 6 wk (follow-up) of CON or INT. Means ± normalized CI are shown. The CI values have been corrected to remove interindividual variation (22). †A main effect of day (i.e., baseline vs follow-up for both groups; *P* ≤ 0.001). *A day–group interaction (*P* ≤ 0.001). #Values are different pre–post within the INT group (*P* ≤ 0.001). There were moderate effect sizes between groups of *d* = 0.68 and 0.61 for V˙O_2peak_ (A) and workload (B), respectively.

Functional capacity in terms of V˙O_2peak_ (Fig. 2A) and power output (Fig. 2B) significantly increased over time (*P* ≤ 0.001), with evidence of a strong interaction effect (*P* ≤ 0.001). The INT group significantly increased (*P* ≤ 0.001) V˙O_2peak_ (3.4 mL·kg^−1^·min^−1^; 95% CI, 2.4–4.3 mL·kg^−1^·min^−1^) and peak power output (19 W; 95% CI, 14–23 W), whereas these outcomes remained unchanged in the CON group.

#### Metabolic regulation

Markers of metabolic regulation at baseline and follow-up are summarized in Table 3. Changes in fasting serum insulin concentrations and the homeostasis model assessment of insulin resistance (HOMA2-IR) were different between the two groups (*P* ≤ 0.044). The INT group significantly decreased fasting serum insulin concentrations (−12.7 pmol·L^−1^; 95% CI, −24.0 to −1.4 pmol·L^−1^; *P* ≤ 0.031) and HOMA2-IR (−0.24; 95% CI, −0.45 to −0.02; *P* ≤ 0.035), whereas these outcomes were unchanged in the CON group. There was a trend for an interaction effect (*P* = 0.066) for homeostasis model assessment of pancreatic β-cell function (HOMA2-β). Fasting plasma glucose and measures derived during the OGTT (composite insulin sensitivity index (C-ISI_Matsuda_), plasma glycemic response, and serum insulinemic response) were not different over time (all *P* ≥ 0.6), with no interaction effects (all *P* ≥ 0.3).

**TABLE 3:**
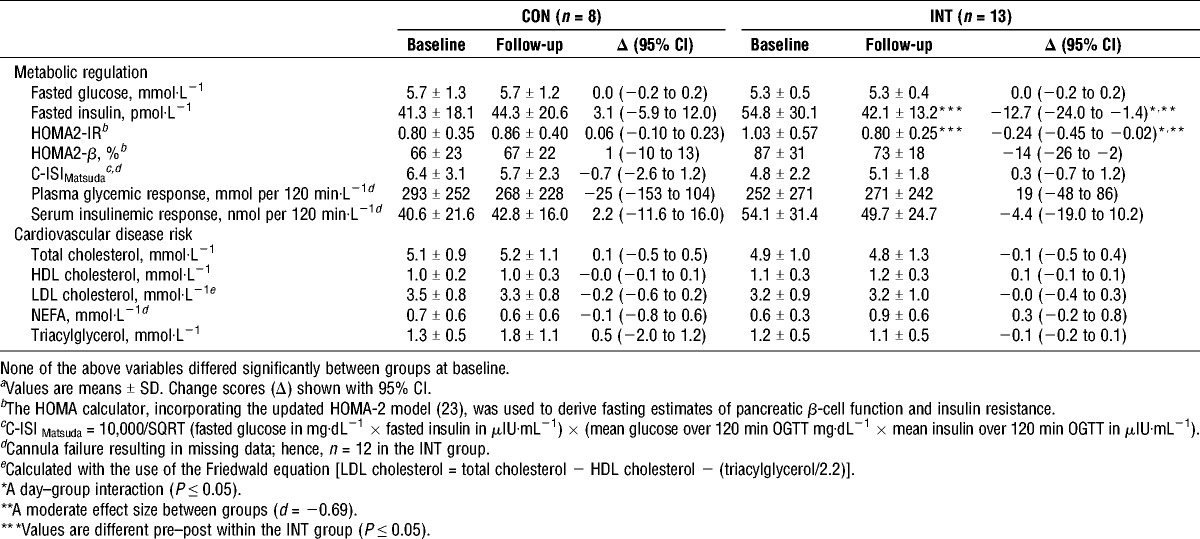
Markers of metabolic regulation and cardiovascular disease risk measured at baseline and after 6 wk (follow-up) of CON or INT*^a^*.

#### Cardiometabolic component risks

Biomarkers of cardiovascular disease risk (Table 3) did not respond differently to 6 wk of INT or CON (all interactions; *P* > 0.3), although there was a trend for an interaction effect (*P* = 0.054) for serum triacylglycerol concentration. Within the INT group, mean Δ change values for total cholesterol and LDL cholesterol increased in participants with injury level of ≥T6 (0.35 and 0.26 mmol·L^−1^, respectively) and decreased in the <T6 group (−0.29 and −0.23 mmol·L^−1^, respectively). Despite nonsignificant interaction effects between these subgroups (*P* > 0.10), standardized effect sizes (*d*) were −0.51 and −0.49 for total cholesterol and LDL cholesterol, respectively.

#### Adipose tissue gene expression

**FIGURE 3:**
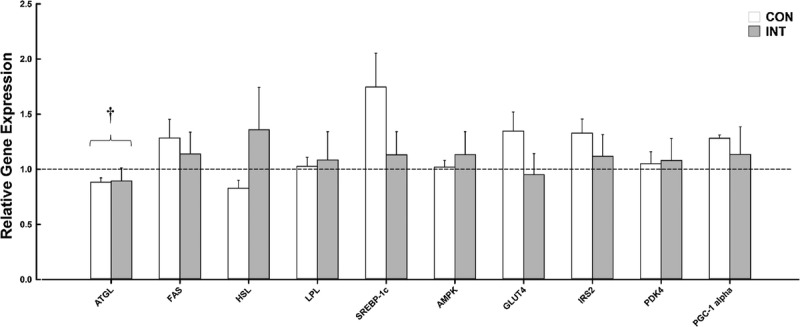
Relative gene expression of 10 key metabolic genes measured in adipose tissue at baseline and follow-up for the CON group (*n* = 8) and the INT group (*n* = 12). *Dashed line* represents no change. Data normalized to PPIA, baseline, and internal calibrator. Sufficient RNA could not be obtained to carry out gene expression analysis for one participant follow-up sample (small adipose tissue yield); hence, *n* = 12 in the INT group. Values are means ± SEM. †A main effect of day (i.e., baseline vs follow-up for both groups; *P* ≤ 0.05).

Gene expression data are presented in Figure 3. ATGL was down-regulated over the course of 6 wk (day effect; *P* = 0.038). There were no interaction effects (all *P* ≥ 0.08) for any of the genes of interest.

## DISCUSSION

Six weeks of home-based moderate-intensity arm-crank exercise in inactive persons with chronic paraplegia significantly improved (i) functional capacity (V˙O_2peak_ and power output), (ii) fasting insulin concentrations, and (iii) HOMA-IR compared with the CON. Home-based exercise successfully provided a considerable volume of structured exercise (180 min·wk^−1^). Reduced insulin resistance was achieved with a relatively modest increase in energy expenditure (↑109 ± 85 kcal·d^−1^) and without concurrent improvements in body composition.

Recent hand-cycle exercise training studies (1,18) have demonstrated similar mean improvements in fasting insulin concentrations, after 6- and 16-wk interventions in individuals with SCI, to those reported herein. Although not calculated using the updated HOMA2 model, these studies also showed improvements in HOMA-IR. The only participant with HOMA-IR above the cutoff (1.65) indicative of metabolic syndrome in persons with SCI (Academy of Spinal Cord Injury Professionals Educational Conference and Expo poster, Hobson et al., 2016) provides an illuminating case series. This participant displayed considerable improvements from 2.72 at baseline to 1.50 at follow-up (Δ − 1.21). A smaller volume of upper-body moderate-to-vigorous aerobic exercise (40 min min·wk^−1^) has recently been shown not to improve vascular health or biomarkers of cardiovascular disease for 16 wk (8). In light of the current study’s findings, it is possible that a greater volume of upper-body exercise (than that proposed by the PAG-SCI) might be necessary to promote beneficial adaptations in insulin resistance.

There is a surprising paucity of research comparing dynamic glucose and insulin responses after arm-crank exercise training in this population. However, changes in insulin sensitivity after 16 wk of an arm-crank ergometry of 5 d·wk^−1^ were recently observed (14). High (70%–80% heart rate reserve (HRR)) and low (40%–50% HRR) intensity arm-crank ergometry have been shown to have detrimental or no effect on insulin sensitivity, assessed using the HOMA–continuous infusion of glucose with model assessment test (6). These findings agree with measures derived from OGTT data in this study, where no significant interaction effect was evident. It has been suggested that insulin sensitivity indices (i.e., ISI_Matsuda_), derived from an OGTT, predominantly represent peripheral insulin sensitivity (23), whereas HOMA-IR reflects hepatic insulin sensitivity (29). Therefore, these data could infer that moderate-intensity upper-body arm-crank ergometry improves hepatic but not whole-body insulin sensitivity. One explanation for improvements in hepatic insulin sensitivity with exercise alone could be a reduction in intrahepatic fat (32). Although a reduction in intrahepatic fat can be achieved irrespective of weight loss (17), such responses have not been observed over such a short duration of exercise training (i.e., 6 wk). Future research should therefore aim to elucidate the mechanisms whereby upper-body exercise improves hepatic insulin sensitivity in persons with SCI.

Convincing evidence that functional electronic stimulation–leg cycling ergometry improves insulin sensitivity, determined from OGTT (ISI_Cederholm_) (5) and gold-standard hyperinsulinemic–euglycemic clamp, is beginning to emerge (15,16,24). In nondisabled individuals, it is well documented that exercise training improves whole-body insulin sensitivity and glucose homeostasis, with adaptations in skeletal muscle being essential to this, because this tissue is responsible for the majority of glucose utilization (7). Because voluntary exercise is restricted to the smaller upper-body musculature for persons with SCI, a large volume of exercise has a relatively modest effect on whole-body energy expenditure and associated weight loss (−1.1 ± 1.7 kg). Therefore, training adaptations specific to these smaller exercising muscle groups might not provide a sufficient stimulus to overcome insulin resistance in other peripheral tissues (i.e., leg skeletal muscle and adipose tissue). For example, it was recently demonstrated that arm-crank ergometry had no effect on protein expression changes of GLUT4, PGC-1α, and AMPK in paralyzed vastus lateralis muscle (14). It therefore seems that skeletal muscle adaptations to exercise are somewhat localized and limited to the muscles activated during exercise training.

Dyslipidemia was common in our participants; 43% had total cholesterol values of ≥5 mmol·L^−1^, 57% had elevated LDL cholesterol (≥3 mmol·L^−1^), and 62% had depressed HDL cholesterol (≤1.03 mmol·L^−1^ for men and ≤1.29 mmol·L^−1^ for women). No significant differences were observed for total cholesterol, LDL cholesterol, or HDL cholesterol. Although a systematic review (4) concluded that there was insufficient evidence suggesting that exercise alone improves dyslipidemia in people with SCI, exercise at a higher-intensity might be necessary to improve lipid profiles in this population (6,25). Within the INT group, participants were stratified into two subgroups on the basis of injury level. It was thought that those with higher-level injuries (≥T6) would have a greater degree of functional impairment at baseline and thus potentially benefit more from the INT. Mean Δ change values responded differently between the two subgroups for total cholesterol and LDL cholesterol, surprisingly, improving in those with an injury level of <T6 and worsening in the ≥T6 group. Although these finding were nonsignificant and based on a small subgroup analysis, they are opposite to hypothesized and posit an interesting message that persons with higher levels of injury/impairment may not adapt in the same way to exercise, perhaps because of other biological limitations (i.e., loss of sympathetic innervation). It has been demonstrated that inflammatory cytokines and concentrations of epinephrine did not change in persons with tetraplegia but were significantly elevated after an acute bout of exercise in able-bodied controls and persons with an SCI of <T6 (intact sympathetic innervation) (27). Cytokines, such as interleukin 6, are released from skeletal muscle in response to exercise (termed *myokines*) and play an important role in regulating whole-body metabolism (28). Therefore, loss of sympathetic innervation of the adrenal medulla may diminish the metabolic responses to exercise in persons with an SCI of ≥T6. Future studies are necessary to confirm this.

To our knowledge, this is the first study to investigate the expression of key genes within subcutaneous adipose tissue involved in glucose and lipid metabolism in inactive persons with chronic paraplegia. Across the 10 genes of interest, no significant between-group responses were evident at the end of the 6-wk period. In contrast, animal models have shown that an exercise training stimulus can increase the expression of genes involved in a variety of metabolic regulation and mitochondrial biogenesis pathways (33,35). It is noteworthy that some of these metabolic adaptations within adipose tissue are independent of body mass changes (34). Because of the reduced energy expenditure, cardiovascular strain, and whole-body metabolic stress associated with upper-body exercise in persons with SCI (25), it is extremely difficult to generate a considerable energy deficit through exercise alone. It is therefore surprising that there is a lack of research looking at the effect of dietary restriction alone, or in combination with exercise, on cardiometabolic component risks in this population. We encourage future work to investigate this, especially because a recent study has shown improvements in whole-body and adipose tissue metabolism for as little as 3 wk with moderate weight loss (−2.4 kg), achieved through a combination of exercise and dietary restriction in nondisabled overweight men and postmenopausal women (38).

V˙O_2peak_ and workload were significantly elevated in the INT group by 17% (Δ, 3.4 mL·kg^−1^ ·min^−1^) and 20%, respectively. These improvements in cardiorespiratory fitness are greater than those observed (~10%) with a smaller volume of moderate-intensity arm-crank exercise (~90 min·wk^−1^) during a longer duration (12 wk) (10,30). A 3-mL·kg^−1^·min^−1^ improvement in cardiorespiratory fitness has been associated with greater than 15% and 19% reductions in all-cause and cardiovascular disease mortality in nondisabled individuals (20). Although only measured for a week at baseline and before follow-up, this is the first study to measure changes in free-living behaviors (PAEE and energy intake) in response to an exercise intervention in this population. At a group level, the increase in estimated free-living PAEE (~109 kcal·d^−1^) measured during the final week of the intervention is similar to the increase in PAEE (~98 kcal·d^−1^) estimated from baseline data_._ However, there was considerable interindividual variability in physical activity responses from baseline to follow-up in the intervention group (Δ in PAEE ranging from −60 to 109, Δ in moderate-to-vigorous physical activity ranging from −4 to 32 min·d^−1^). It is conceivable that for *some* participants, the prescribed exercise intervention simply replaced nonprescribed (existing) physical activity of a similar intensity. This concept has been referred to as “substitution” and may erode predicted adaptations and observed changes in various outcome measures (i.e., body mass) (37). However, 67% of the INT group performed more PAEE than predicted, which may indicate that substitution of behaviors was not as prevalent in this previously inactive SCI cohort.

The main strength of the current study is the use of a validated multisensor device, which permitted the screening of inactive participants at baseline and the analysis of novel concepts (i.e., substitution of physical activity) that had not been looked at previously in persons with SCI. Every attempt was made to recruit a relatively homogenous sample (i.e., inactive, chronic paraplegia) with elevated cardiometabolic component risks. Although participants were inactive, careful assessment of lipid and glycemic profiles suggests that this random sample of participants presented with fairly normal cardiovascular disease risk profiles at baseline. Although this may have resulted in either basement or ceiling effects, lipid profile responses within the INT group did not respond differently when participants were stratified into either high or low risk on the basis of the National Cholesterol Education Program Adult Treatment Panel III guidelines. To ensure a more homogenous sample, more stringent inclusion criteria could have been applied. However, this would likely have negatively affected recruitment, which is an ongoing challenge for researchers working with this population. As such, this is one of the largest studies to assess the effect of moderate-intensity arm-ergometry exercise on cardiometabolic component risks in persons with SCI. Compared with previous research that has predominantly focused on male participants, 29% of our cohort was female, which better reflects the sex distribution in the wider population (US national statistics database).

## CONCLUSIONS

In summary, our study shows that 6 wk of home-based moderate-intensity arm-crank ergometry in inactive adults with chronic paraplegia induced positive changes in functional capacity and fasting insulin concentrations, as well as improvements in hepatic insulin sensitivity. There were no changes over time or differences between groups in markers of peripheral insulin sensitivity, adipose tissue metabolism, or other biomarkers of cardiovascular disease risk. Although this intervention led to a modest increase in PAEE, we observed beneficial metabolic adaptations, which seem to be associated with improved liver function.
